# Supplementary material for: Metaplasia creates a permissive niche for select Fusobacterium species in the stomach
Source: mBio. 2026 Oct 9;17(11):e01747-26. doi: 10.1128/mbio.01747-26 (PMC13753815; doi:10.1128/mbio.01747-26)
Supplement: Supplementary file 1 [file mbio.01747-26-s0001.pdf]

Supplementary Material

Metaplasia Creates a Permissive Niche for Select *Fusobacterium* Species in the Stomach

Gómez-Garzón, C.; Chen, Q.; O'Brien V. P.; Garman, K.S.; Salama, N. R.

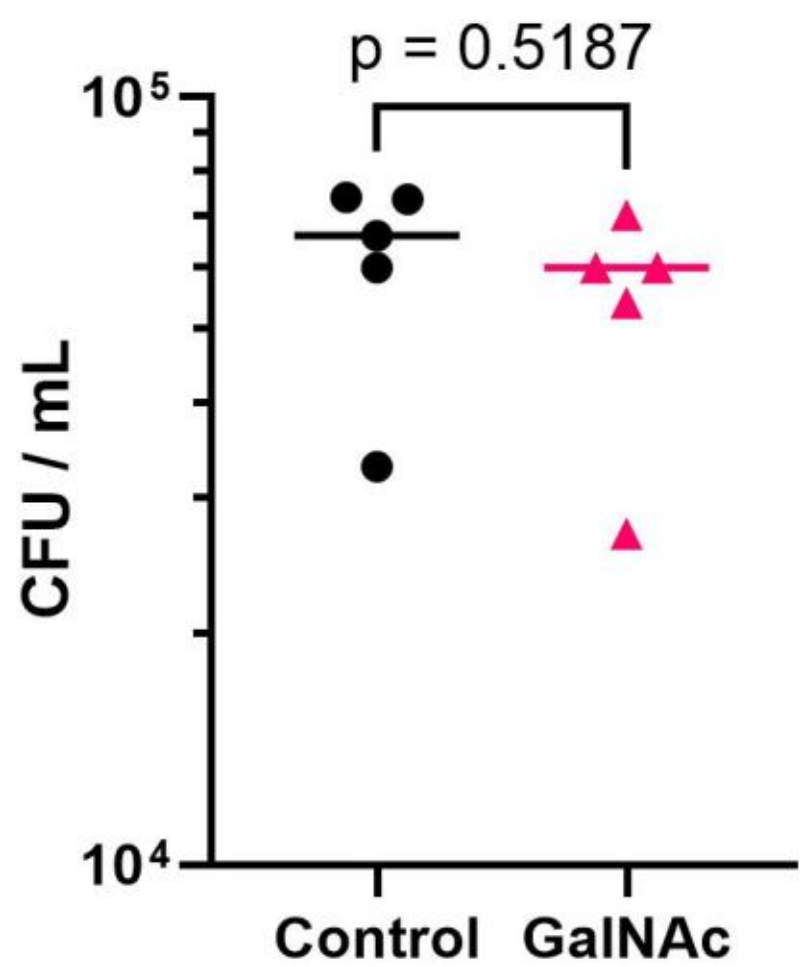

Supplementary Figure S1. GalNAc does not affect the viability of *F. animalis*. *F. animalis* CFU counts after 30 min incubation in 10:1 DMEM10 / BB10 medium supplemented with either 25 mM GalNAc or the equivalent amount of PBS (Control). These values correspond to independent experiments. Cell suspensions were prepared to achieve cell densities of ~10^4 CFU/mL, corresponding to an MOI of 0.1 in co-cultures with AGS cells. The *p*-value corresponds to an unpaired, two-tailed t-test between groups.

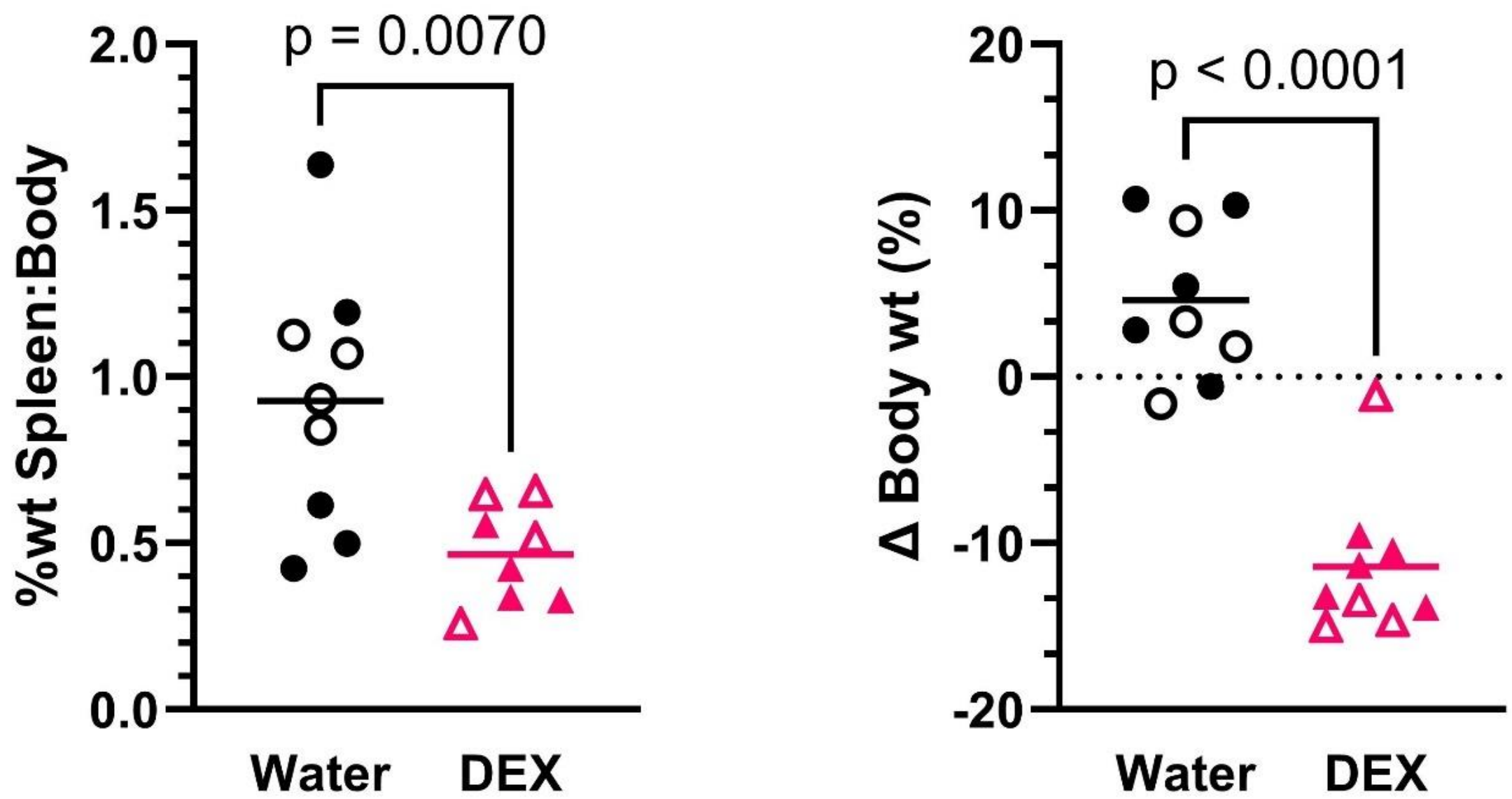

Supplementary Figure S2. Body changes in mice treated with oral dexamethasone. Comparisons were made between the control group that received non-acidified, autoclaved drinking water (Water) and the treatment group that received drinking water supplemented with 1 mg/L dexamethasone *ad libitum*. (DEX) **A)** Body weight (wt) percentage represented by the spleen at the end of the experiment. **B)** Percentage change in body weight between the start and the end of the experiment. Horizontal lines show the mean of each group, and *p*-values were calculated from non-paired, two-tailed t-tests. The data correspond to mice from the experiment shown in Fig. 4 of the main text and were obtained from two independent experiments indicated by filled and open symbols.

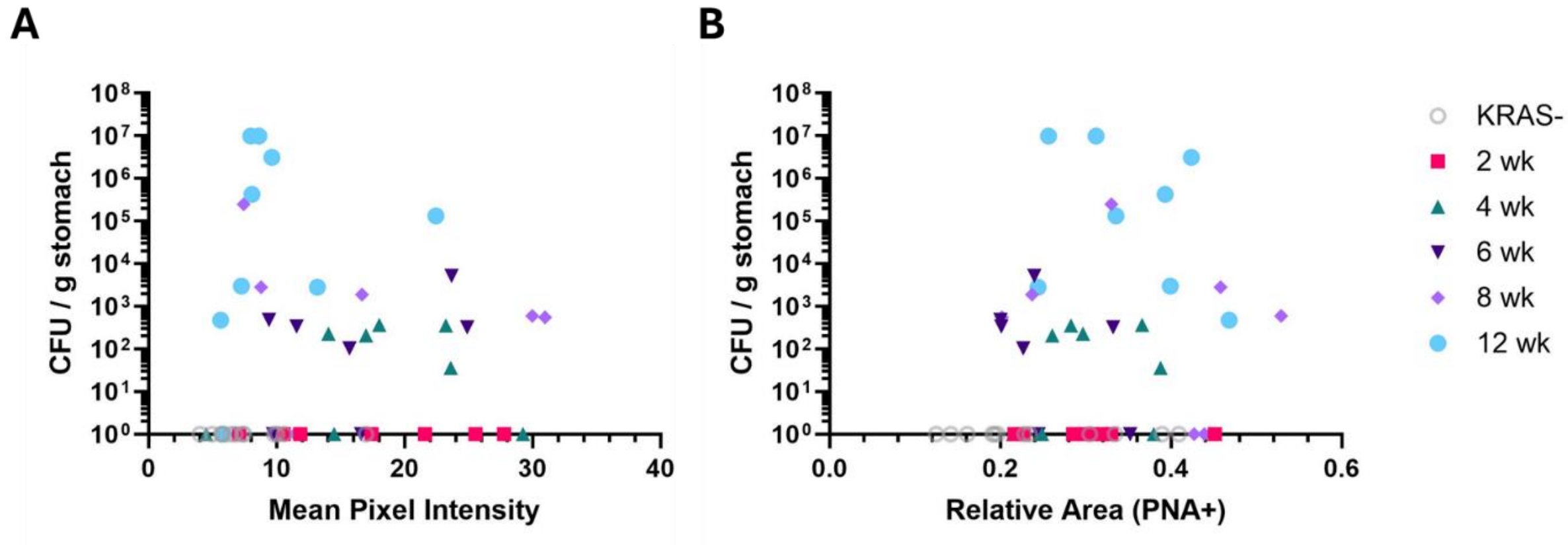

Supplementary Figure S3. Gastric *F. animalis* loads show no correlation with Gal-GalNAc expression in the stomach of the GIM mouse model during Kras-driven preneoplastic progression. Gal-GalNAc display was quantified as **A)** Mean Pixel Intensity (linear regression: R^2 = 0.027, p = 0.25, semi-log regression: R^2 = 0.001, p = 0.82) and **B)** Relative Area of PNA+ Cells (R^2 = 0.0001, p = 0.94, semi-log regression: R^2 = 0.043, p = 0.15) as shown in **Fig. 5** of the main text. Plotted data were obtained from two independent experiments (except for the 12-week timepoint) and colored according to the weeks (wk) post-induction, from 2 to 12 weeks, representing different points of the preneoplastic progression.

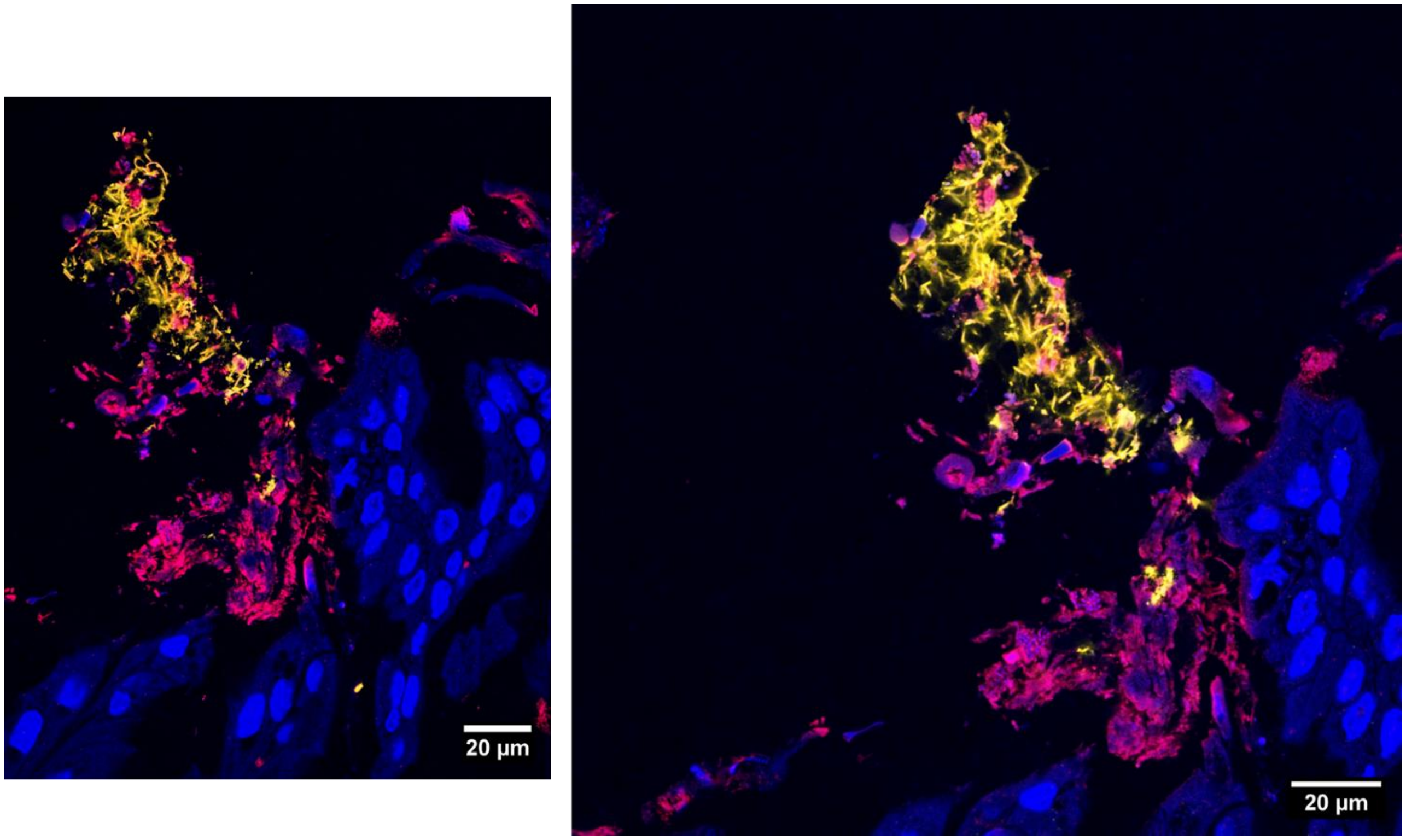

Supplementary Figure S4. Magnification of a *Fan*-positive spot detected in a mFISH assay with a *Fusobacterium* 23S rRNA probe. These images correspond to magnifications of the left panel of Fig. 8 in the main text. The foci labeled with the *Fusobacterium* probe (yellow) exhibits the classical spindle-like cell shape and size (2 – 10 µm) expected for *Fan*. Signal in red corresponds to Eubacteria 16S rRNA probe. Mouse tissue nuclei were labeled with DAPI (blue).

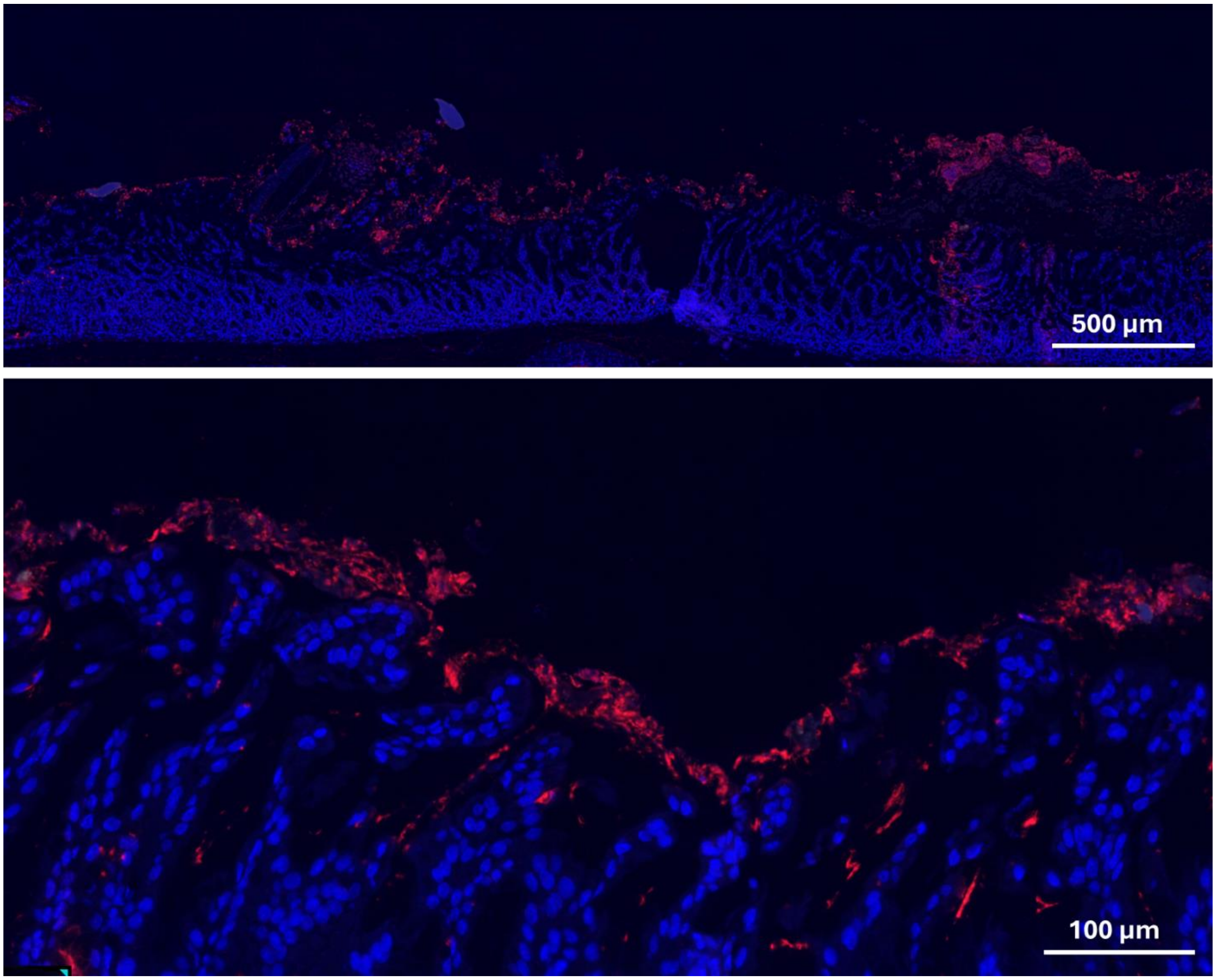

Supplementary Figure S5. Control tissue for the mFISH assay shown described in Fig. 8 in the main text. Both panels correspond to gastric tissue (corpus) of two representative mock-infected *Kras*+ mice labeled with the *Fusobacterium* 23S rRNA (yellow) and Eubacteria 16S rRNA (red) probes. Mouse tissue nuclei were labeled with DAPI (blue). No *Fan*-positive foci were detected in these samples, while similar patterns of Eubacteria-positive foci and gland atrophy were observed.

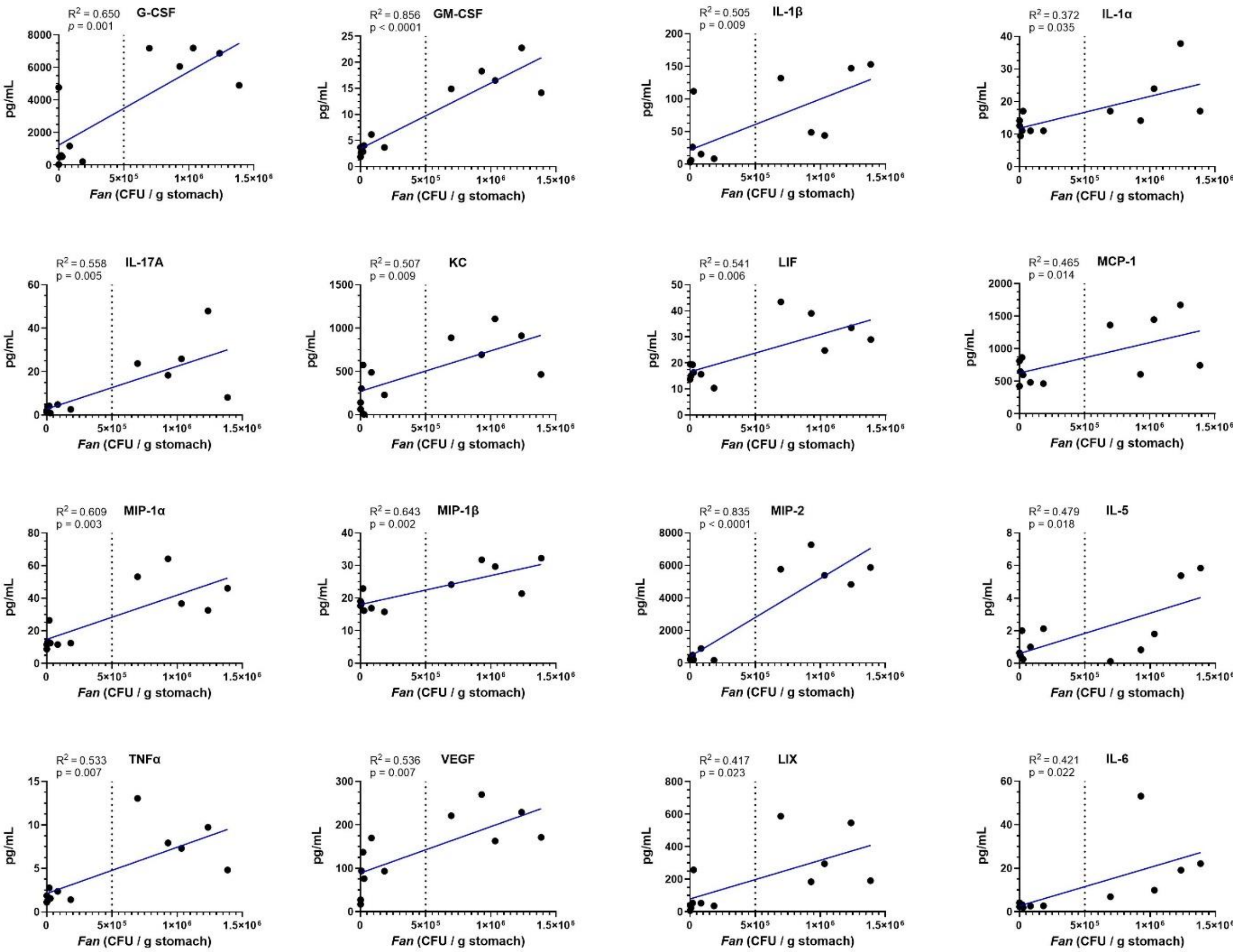

Supplementary Figure S6. **Linear regressions for cytokine concentration *vs. F. animalis* (*Fan*) titers for those cytokines with a significant difference between mock and infected groups.** The R^2 corresponds to the coefficient of determination, and the *p*-values were calculated from F tests for the slope equaling zero. The vertical dotted lines indicate the cutoff value (5×10^5 CFU/g) used to define the low and high infection groups. Plotted data correspond to the experiment shown in Fig. 9 in the main text.

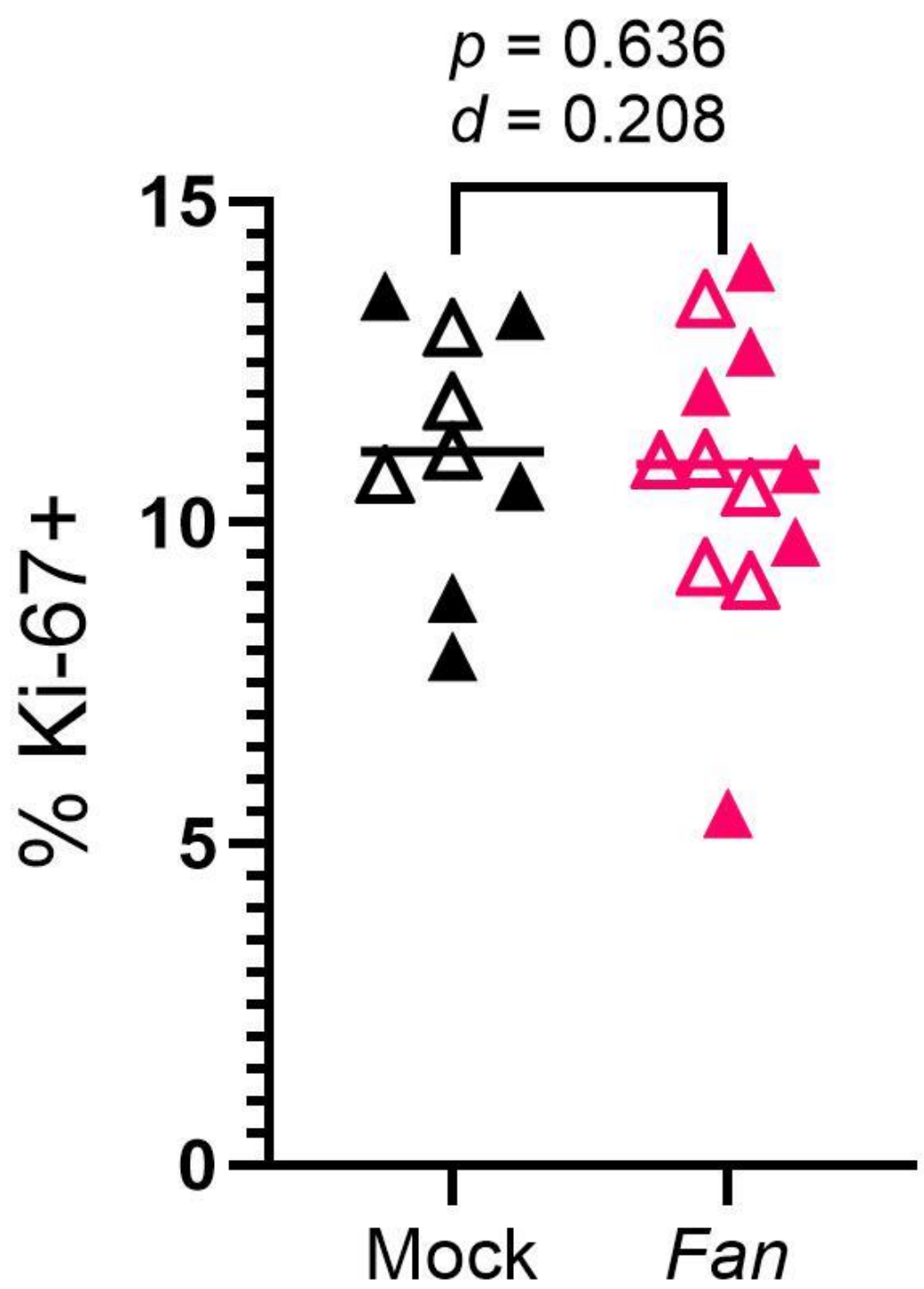

Supplementary Figure S7. **F. animalis does not alter cell proliferation in the *Mist1-Kras* mouse model.** Comparison of Ki-67+ cells in the corporal epithelium of *Kras*+ mice infected for 6 weeks with *F animalis* SB010 (*Fan*) and a mock-infected control (Mock) group. The percentage of Ki-67+ cells in the corpus was calculated by image analysis of immunofluorescence images using the STARDIST pipeline. The *p*-value corresponds to an unpaired, two-tailed t-test between groups, and *d*, to the corresponding Cohen's d value for the effect size.

Supplementary Table S1. R^2 and *p*-values of the linear regressions of those quantified cytokines for which the null hypothesis of equal means could not be ruled out with α = 0.05 by an ANOVA test.

Cytokine	*p*-value	R^2
**Eotaxin***	0.022	0.424
**IFNy**	0.822	0.005
**IL-2**	0.904	0.001
**IL-3**	0.137	0.208
**IL-4**	0.087	0.265
**IL-5****	0.018	0.479
**IL-7**	0.356	0.086
**IL-9**	0.303	0.105
**IL-10**	0.375	0.079
**IL-12P40**	0.397	0.072
**IL-12P70**	0.229	0.141
**IL-15**	0.656	0.021
**IP-10**	0.108	0.237
**M-CSF**	0.171	0.179
**MIG**	0.136	0.208
**RANTES**	0.918	0.001

* ANOVA *p*-value = 0.069, F = 3.19
** ANOVA *p*-value = 0.146, F = 2.22

Supplementary Table S2. *Fusobacterium* strains used in this study,

***Fusobacterium* strain**	**Source**	**Additional information**
F. nucleatum ATCC 23726	Donated by Chenggang Wu	Reference ATCC strain
F. nucleatum ATCC 25586	Donated by Jeffrey McLean	Reference ATCC strain
F. animalis SB010	Donated by Susan Bullman	Isolated from a colorectal cancer case (1)
F. animalis 7_1	Donated by Emma Allen-Vercoe	Isolated from a Chron's disease case (2)
F. animalis CG01	This study	Gastric clinical isolate
	Isolated from an antral gastric brushing of a 60 – 70 years old Black/non-Hispanic female with history of treatment for *H. pylori* infection and no evidence of GIM at the time of the collection.	WGS uploaded in the NCBI Genome database under the accession number JCACXA000000000
F. periodonticum pool	This study	Gastric clinical isolates
	Isolated from antral and gastric body brushings of a 60 – 70 years old Indian/non-Hispanic male with history of treatment for *H. pylori* infection.	Representative 16S rDNA sequence uploaded to the NCBI GenBank database under the accession number PZ585466
F. polymorphum 12230	Donated by Jeffrey McLean	Trans-tracheal clinical isolate (3)

References

1. Bullman S, Pedamallu CS, Sicinska E, Clancy TE, Zhang X, Cai D, Neuberg D, Huang K, Guevara F, Nelson T, Chipashvili O, Hagan T, Walker M, Ramachandran A, Diosdado B, Serna G, Mulet N, Landolfi S, Ramon S, Fasani R, Aguirre AJ, Ng K, Élez E, Ogino S, Tabernero J, Fuchs CS, Hahn WC, Nuciforo P, Meyerson M. 2017. Analysis of *Fusobacterium* persistence and antibiotic response in colorectal cancer. Science (1979) 358:1443–1448. https://doi.org/10.1126/science.aal5240

2. Strauss J, Kaplan GG, Beck PL, Rioux K, Panaccione R, Devinney R, Lynch T, Allen-Vercoe E. 2011. Invasive potential of gut mucosa-derived *Fusobacterium nucleatum* positively correlates with IBD status of the host. Inflamm Bowel Dis 17:1971–1978. https://doi.org/10.1002/ibd.21606

3. Han YW, Shi W, Huang GTJ, Kinder Haake S, Park NH, Kuramitsu H, Genco RJ. 2000. Interactions between periodontal bacteria and human oral epithelial cells: *Fusobacterium nucleatum* adheres to and invades epithelial cells. Infect Immun 68:3140–3146. https://doi.org/10.1128/iai.68.6.3140-3146.2000
